# Case of Complete Occlusion of the Vagina, Consequent upon an Abortion

**Published:** 1875-11

**Authors:** Braxton Hicks


### Case of Complete Occlusion of the Vagina and Retention of the Menses, Consequent upon an Abortion—Relief by Operation.

Under the Care of Dr. BRAXTON HICKS and Dr. GALABIN.

Mrs. G., aged twenty-eight, aborted about the middle of the fourth month of her first pregnancy, in October, 1874. No interference was necessary to remove the ovum. She was confined to her bed for many weeks after the abortion, and suffered much abdominal pain. When convalescent, she discovered for the first time that some obstruction had been produced in the vagina. No vaginal examination had been made by her attendant since the time of abortion. In February, 1875, she began to suffer from spasmodic pain in the abdomen, no menstrual discharge having in the meantime appeared. In March she again had recourse to her medical attendant, who passed some instrument (apparently a metal catheter), by means of which a few small clots were liberated. This was repeated several times, but the pain was not relieved, and in June an attempt was made to restore the occluded canal by separating the tissues with blunt instruments and by the fingers.

No anesthetic was given. On this occasion no menstrual fluid was liberated. About a week after the operation the feces began to pass by the vagina, and this continued up to the time of her admission.

She was admitted into Guy's Hospital on August 25th. The vagina was found to be completely occluded, and converted into a shallow pouch, at the bottom of which was a smooth opening into the rectum, admitting one finger very easily. For the last month the pain had come on only once a day. It was violent in character, and lasted for six or seven hours.

On August 30th Dr. Braxton Hicks commenced the operation for the restoration of the vagina, no anesthetic being given. The cicatricial tissue was carefully torn apart with a blunt knife by the aid of the finger. Some rather considerable bleeding occurred, and when some advance had been gained the operation was, for the time, suspended, oiled lint being placed in the wound. The patient continued to have paroxysmal pain each day, but it was subdued for the time by the injection of $\frac{1}{8}$ grain of acetate of morphia subcutaneously.

On September 27th, in the absence of Dr. Braxton Hicks, the operation was resumed by Dr. Galabin. The vagina at this time was still a *cul de sac*, barely an inch and a half deep, bounded by very dense cicatricial tissue. Chlroform was given upon this occasion. The fundus uteri could then be felt, somewhat retroverted, but reaching as high as the umbilicus. On being tilted forward, it could be seen to contract powerfully from time to time. A transverse incision was made through the superficial cicatrix, and a passage was then gradually torn by a blunt instrument, a catheter being kept in the uretha. A large cavity was at length reached, having smooth walls. It appeared to consist in the main of the distended uterus, but no os or cervix could be detected. The opening was enlarged to a size admitting two fingers, and about fifteen ounces of dark, treacly fluid (not offensive) were expelled. A pad and bandage were then applied. The same fluid continued to flow away in some quantity until about midnight, but ceased from that time. The evening temperature was 100°, and the pulse 100, but the patient suffered no inconvenience, and from the following day pulse and temperature were normal. The next morning the cavity was injected with a weak solution of permanganate of potash, which brought away much grumous blood. This injection was repeated twice a day.

The opening which had been made showed a great tendency to contract, and on October 2d its dilatation was commenced by the smallest-sized Barnes' dilating bag. This was introduced for a few hours each day, and on the 4th was replaced by the medium-sized bag. On the 8th the canal was so far dilated that the bag could with difficulty be retained. The upper part of the cavity within had now much contracted, and its fundus could just be reached by the examining finger. At a distance of 1¼ inch below the fundus was felt a firm ring of apparently muscular tissue, the length of the whole cavity being 2¾ inches. Its lower part showed little tendency to contract, and was made up partly of a pouch extending backward and to the right. Nothing like an external os or cervix uteri could be detected.

The patient still continues nearly in this condition, and the recto-vaginal fistula remains for further operative treatment.

REMARKS.—The operation in this case presented some difficulty on account of the close adhesion which had been formed at the lower part of the cicatrix, between the rectum and the bladder, so that scarcely more than a quarter of an inch of dense tissue appeared to intervene between the rectum and a catheter in the urethra. On this account the recto-vaginal fistula had unfortunately resulted from the first attempt at operation before the patient's admission into the hospital. It was also impossible to discover the position of the cervix uteri, and this would seem indeed to have been entirely destroyed or blended with cicatricial tissue. The shape and the behavior of the lower part of the lower part of the distended cavity appeared to show that it was not composed of uterine tissue, and the firm ring afterwards felt at a distance of 1¼ inch from the fundus seemed to correspond to the internal os uteri. The cavity was, therefore, probably made up partly of a remnant of the distended body of the uterus, and partly of a remnant of the upper part of the vagina.—*Obstetrical Journal.*

